# Impact of a Better Persistence with Antihypertensive Agents on Ischemic Stroke Outcomes for Secondary Prevention

**DOI:** 10.1371/journal.pone.0065233

**Published:** 2013-06-11

**Authors:** Jie Xu, Xingquan Zhao, Yilong Wang, Chunxue Wang, Liping Liu, Baoying Sun, Anxin Wang, Yongjun Wang

**Affiliations:** 1 Department of Neurology, Beijing Tiantan Hospital, Capital Medical University, Beijing, China; 2 Jinan Central Hospital Affiliated to Shandong University, Jinan, Shandong, China; Cardiff University, United Kingdom

## Abstract

**Background:**

The efficacy of antihypertensive (AH) treatment after stroke has been investigated in several randomized clinical trials. However, non-adherence to AH medication is common for stroke patients in “real world” setting. The purpose of this study was to assess the impact of persistence with AH agents on ischemic stroke (IS) outcomes.

**Methods and Results:**

Using the China National Stroke Registry, we analyzed data from 8409 IS patients with hypertension. Persistence with AH therapy (high persistence ≥75%, low persistence <75%) was measured by patient self-report at 3, 6, and 12 months after stroke. Multivariate logistic regression model was used to assess the relationship between persistence and IS outcomes (stroke recurrence, combined vascular events and death) at 12 months. Of the 8409 patients in this study, 40.0% were female and the mean age at study entry was 66.7 years. 31.6% of patients had high persistence with AH drugs, and 68.4% had low persistence during 1 year after stroke onset. High persistence with AH drugs significantly decreased the risk of stroke recurrence (odds ratio, 0.78; 95% CI, 0.68 to 0.89), combined vascular events (0.71; 0.63–0.81) and death (0.44; 0.36–0.53) compared with low persistence.

**Conclusions:**

Our study reinforces the benefits of AH medications in routine clinical practice and highlights the importance of persistence with AH therapy among IS patients known to be hypertensive within the first year of an event.

## Introduction

The efficacy of antihypertensive (AH) treatment after stroke has been investigated in several randomized clinical trials (RCTs) [1]–[7]. Current American Heart Association/American Stroke Association (AHA/ASA) guidelines advocate blood pressure reduction as a class IA recommendation in patients who have had an ischemic stroke (IS) or transient ischemic attack (TIA). Perindopril Protection Against Recurrent Stroke Study (PROGRESS) showed that, active treatment reduced the risk of total major vascular events by 26% (95% confidence interval [CI]: 16%–34%) and stroke risk by 43% (95% CI: 30%–54%) [5]. A meta-analysis [6], included 7 randomized trials performed through 2002, showed that treatment with AH drugs was associated with significant reductions in recurrent strokes (relative risk [RR], 0.76; 95% CI: 0.63 to 0.92), myocardial infarction (RR, 0.79; 95% CI, 0.63 to 0.98), and all vascular events (RR, 0.79; 95% CI, 0.66 to 0.95). Another systematic review conducted in 2009 [7] showed that, across 10 trials, the odds ratio for the prevention of stroke recurrence by blood pressure lowering was 0.78 (95% CI: 0.68–0.90). However, RCT findings on efficacy cannot automatically be expected to represent effectiveness in clinical settings. The adherence to medication in clinical trial settings may not be representative of adherence in “real world” settings [8].

Medication adherence is a growing concern to clinicians and healthcare systems because of mounting evidence that non-adherence is prevalent and associated with adverse outcomes [9]. Little if anything is known about AH adherence and clinical outcome among patients with an initial stroke. The aim of our study was to evaluate the association between persistence with AH therapy and clinical outcomes among IS patients with hypertension for secondary prevention. Data were obtained from the China National Stroke Registry (CNSR), a nationwide, prospective registry that is being used to assess the diagnosis, treatment, and prevention of stroke in China [10].

## Methods

### Ethical Approval, Informed Consent and Patient Privacy

The study protocol was submitted to and approved by the central Institutional Review Board at Beijing Tiantan Hospital. All patients or their designated relatives provided written informed consent and the privacy of patients was strictly protected.

### Study Population

The design, rationale and baseline information of CNSR has been described previously [10]. Briefly, CNSR was a nationwide, hospital-based registry of consecutive stroke patients (≥18 years) within 14 days after stroke onset who were admitted to 1 of 132 participating hospitals in China. The participating hospitals included 100 tertiary and 32 secondary urban hospitals, selected from each of the 27 provinces and 4 municipalities in Mainland China.

In this study, we restricted the study population to IS patients with hypertension. IS was diagnosed according to World Health Organization criteria [11] combined with brain computed tomography or magnetic resonance confirmation. According to JNC 7 criteria [12], hypertension was defined when a patient’s blood pressure was ≥140/90 mm Hg on repeated measurements during the hospitalization or the patient was on AH medication. The information about the prescription of AH medication at discharge was extracted from medical records. The major classes of AH secondary prevention were categorized into: angiotensin-converting enzyme inhibitors, angiotensin receptor blockers, calcium channel blockers, diuretics, and β-blockers.

### Assessment of Persistence

Persistence, which is a type of adherence, is defined as the overall duration of drug therapy [8]. Persistence with AH therapy was assessed through the telephone interview and based on patient self-report. If a patient was incommunicable, we would contact his/her designated relatives. At the 3-, 6-, and 12-month after initial stroke, patients were asked whether they had taken AH therapy since the last follow up. A ‘yes’ answer at the 3- month follow-up was defined as 3 months’ duration of therapy. A ‘yes’ answer at 6-month follow-up indicated that a patient had been taking medicine from 3-month to 6-month. A ‘yes’ answer at 12-month follow-up indicated that a patient had been taking medicine from 6-month to 12- month. Persistence was calculated as the ratio of the cumulative duration of AH therapy and the duration of overall follow-up. In this case,the persistence of each patient can only be 0%,25%,50%,75% or 100%. For example, if a patient had a ‘yes’ answer with AH drugs used at 3 months follow up timepoint, a “no” answer at 6 months and a “yes” answer at 12 months, the persistence level was calculated as (3+0+6)/12 = 75%.

Persistence of ≥75% was defined as high. Persistence <75% was defined as low, including “low persistence” group and “no treatment” group (patients who did not take any AH therapy during the 12-month follow-up period). Death during follow-up was considered as the end of the study and persistence was calculated for the period before death. For example, if a patient died at 3 months follow up timepoint, and had a ‘yes’ answer with AH drugs used at 3 months follow up timepoint by his/her designated relatives, the persistence level was calculated as 3/3 = 100%.

### Outcome Measures

At 12-month after stroke onset, the outcomes of all patients were assessed through telephone follow-up, including recurrence of stroke (re-hospitalization with a diagnosis of new IS or intracerebral hemorrhage), combined vascular events (including stroke recurrence, myocardial infarction, or pulmonary embolism), and death from any cause. The telephone follow-up was conducted centrally for all included patients and was based on a shared standardized interview protocol. If the patients answered they had experienced re-hospitalization for any of the above events, we would contact the hospitals that admitted these patients to verify the diagnosis. All events were based on clear documentation in medical records.

### Covariates

Covariates, thought to influence the outcomes of stroke and the factors associated with persistence, included patient demographic information (gender, age, level of education, monthly household income, types of health insurance, marital status, and living condition), vascular risk factors (history of stroke [defined as a medical chart-confirmed history of stroke, including IS, intracerebral hemorrhage, or subarachnoid hemorrhage], myocardial infarction [defined as a reported history of myocardial infarction or cardiac surgery], atrial fibrillation [defined as a reported history of atrial fibrillation, or diagnosed using the patient’s in-hospital ECG(s)], diabetes mellitus [defined as a reported history of diabetes mellitus, or use of insulin or oral hypoglycemic agents], dyslipidemia [defined with a history of hypercholesterolemia, or use of lipid lowering agents], current or previous smoking [defined as an individual who smoked at the time of stroke or had quit smoking within 1 year], moderate or heavy drinking [≥2 standard alcoholic beverages consumed per day] ), systolic blood pressure at discharge, class of prescribed AH drug at discharge, severity of stroke on admission (National Institutes of Health Stroke Scale, NIHSS [13] ), dysphagia, posterior circulation infarct (classified according to Oxfordshire Community Stroke Project criteria [14]), co-medication at time of discharge, including antiplatelet agents, anticoagulants, lipid-lowering agents and anti-diabetic medications.

### Statistical Analysis

For the descriptive analysis, proportions were used for categorical variables and means with standard deviations were used for continuous variables. Differences between groups were compared using the χ2 test and t test for categorical and continuous variables, respectively. The association between persistence with AH therapy and clinical outcomes (stroke recurrence, combined vascular events and death) were analyzed using multivariable logistic-regression models, which were adjusted for the covariates that showed significant association with AH medication persistence in the univariable analysis (*P*<0.05). Before proceeding to the multivariable regression models, the potential for co-linearity between these covariates was assessed. To identity risk factors independently associated with the stroke outcomes, multivariable logistic regression analysis was performed using backward selection method. The overall significance level for the study was a *P* value of <0.05 using a 2-sided test. Data were analyzed using SAS version 9.1.3 statistical software (SAS Institute, Cary, NC).

## Results

### Patients’ Characteristics

**Figure 1:**
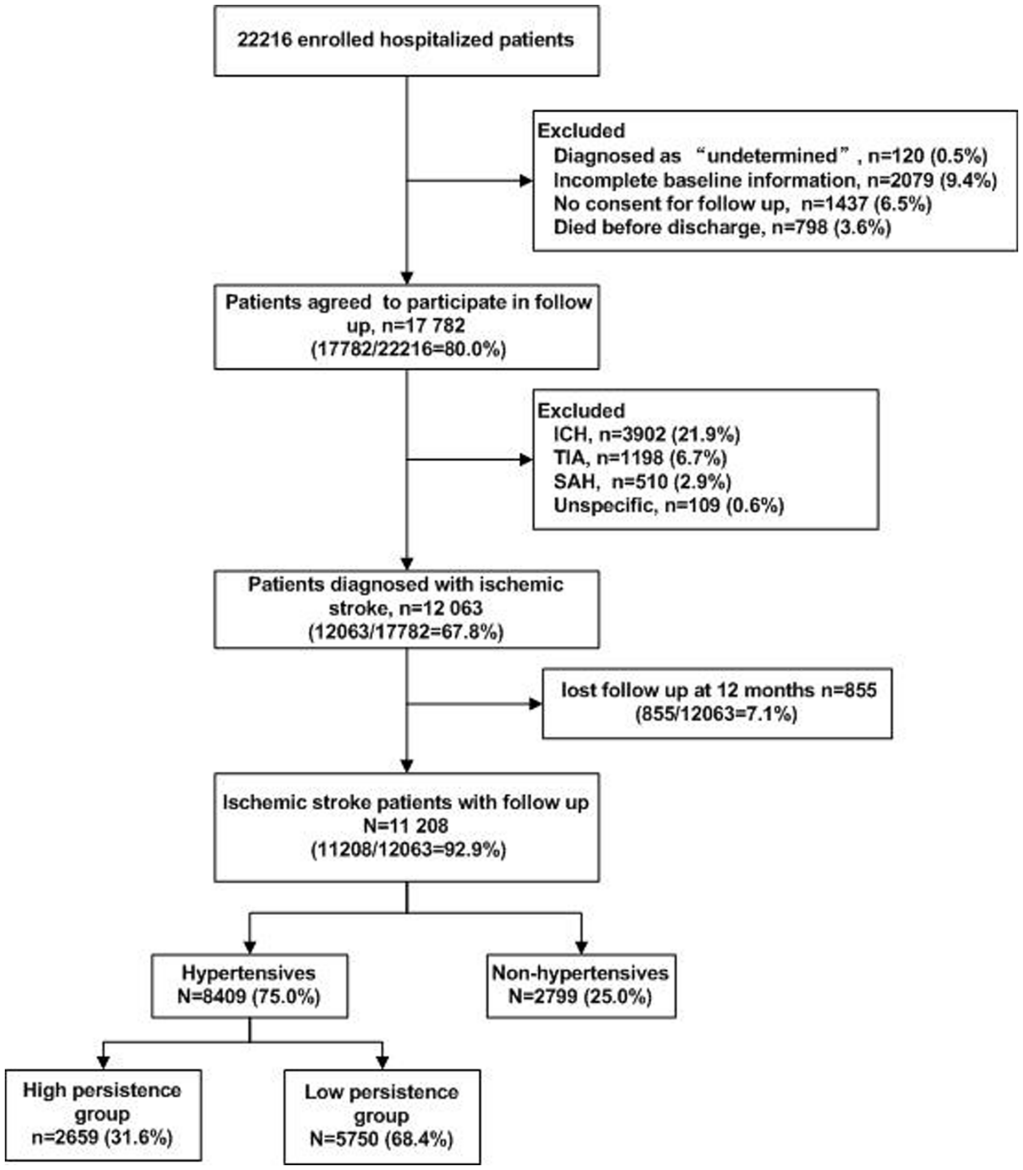
Patient flow diagram. Abbreviations: ICH, Intracerebral Hemorrhage; SAH, Subarachnoid Hemorrhage; TIA, Transient ischemic attack.

Of the 22,216 patients enrolled in the CNSR, 12,063 had a diagnosis with IS and had completed baseline information. Among them, 11,208 IS patients had completed 1 year follow up. Of these, 8,409 were diagnosed with hypertension, who entered into the final analysis in this study (see Figure 1). Of these, 40.0% were female and the mean age at study entry was 66.7 years. About one-third of patients (31.6%) had high persistence (≥75%) with AH drugs, and 68.4% had low persistence (<75%) during 1 year after stroke onset.

Table 1 illustrates baseline characteristics of hypertensive patients with IS according to the level of persistence with AH therapy. There were more patients in the low persistence group who were older, had less education, had vascular risk factors, had dysphagia and had severe symptoms on admission (NIHSS>15), compared with patients in the high persistence group. In contrast, there were more patients in the high persistence group who used other secondary prevention medication (antiplatelet agents, anticoagulants, lipid-lowering agents and anti-diabetic medications) at time of discharge, compared with patients in the low persistence group.

**Table 1:** Baseline characteristics in relation to persistence for identification of predictors of persistence.

Variables	Low persistence group (n = 5750)	High persistence group (n = 2659)	*P value*
**Demographics variables**			
Female	2305 (40.1%)	1059 (39.8%)	0.82
Age			
≤65	2396 (41.7%)	1372 (51.6%)	<0.0001
66∼75	1882 (32.7%)	833 (31.3%)	
≥76	1472 (25.6%)	454 (17.1%)	
Types of health insurance			
Public insurance	4503 (78.3%)	2113 (79.9%)	0.31
Private insurance	188 (3.3%)	76 (2.9%)	
own expense	1059 (18.4%)	460 (17.3%)	
Level of education			
Elementary or below	2784 (48.4%)	1067 (40.1%)	<0.0001
Middle school	1440 (25.0%)	725 (27.3%)	
High school or above	1526 (26.5%)	867 (32.6%)	
Monthly household income			
≤500	614 (10.7%)	276 (10.4%)	<0.0001
500–1000	1272 (22.1%)	649 (24.4%)	
1001–3000	1965 (34.2%)	971 (36.5%)	
≥3000	381 (6.6%)	203 (7.6%)	
Unknown	1518 (26.4%)	560 (21.1%)	
Married*	5113 (89.4%)	2415 (91.1%)	0.02
Live alone#	202 (3.5%)	81 (3.1%)	0.23
**AH medication history**	3609 (62.8%)	1838 (69.1%)	<0.0001
**SBP at discharge, mean (SD), mm Hg**	156.2±24.1	156.9±23.0	0.28
**Class of initially prescribed AH drugs at discharge** †			
ACEI	883 (21.3%)	614 (23.1%)	<0.0001
CCB	2364 (57.0%)	1545 (58.1%)	
ARB	97 (2.3%)	98 (3.7%)	
Diuretics	93 (2.2%)	48 (1.8%)	
Beta-blockers	137 (3.3%)	72 (2.7%)	
More than one AH drug class	572 (13.8%)	282 (10.6%)	
**Co-medication at time of discharge**			
Antiplatelet agents	3600 (62.6%)	2007 (75.5%)	<0.0001
Anticoagulants‡	46 (6.8%)	31 (16.0%)	<0.0001
Lipid-lowering agents	2098 (36.5%)	1194 (44.9%)	<0.0001
Anti-diabetic medications§	1007 (50.8%)	588 (73.2%)	<0.0001
**Vascular risk factors**			
History of stroke	2227 (38.7%)	946 (35.6%)	0.006
Myocardial infarction	1139 (19.8%)	444 (16.7%)	<0.001
Atrial fibrillation	681 (11.8%)	194 (7.3%)	<0.0001
Diabetes mellitus	1984 (34.5%)	803 (30.2%)	<0.0001
Dyslipidaemia	3536 (61.5%)	1660 (62.4%)	0.12
History of smoking	2182 (38.0%)	1051(39.5%)	0.17
History of drinking	527 (9.2%)	229 (8.6%)	0.41
**NIHSS scores at admission**			
0–4	2758 (48.0%)	1474 (55.4%)	<0.0001
5–14	2149 (37.4%)	1019 (38.3%)	
≥15	843 (14.7%)	166 (6.2%)	
**Dysphagia**	580 (10.1%)	232 (8.7%)	0.047
**Posterior circulation infarct**	950 (16.5%)	473 (17.8%)	0.15

Abbreviations: ACEI, angiotensin-converting enzyme inhibitors; AH, antihypertensive; ARB, angiotensin receptor blockers; CCB, calcium channel blockers; SBP, Systolic blood pressure; SD, standard deviation.

* missing 39.

# missing 72.

† based on 6806 patients who were prescribed with AH medication.

‡ based on 875 patients with atrial fibrillation.

§ based on 2787 patients with diabetes mellitus.

### Impact of Drug Class on Persistence with AH Drugs

The mean blood pressure level at hospital discharge was similar between high persistence and low persistence group, however, the proportion of prescribed AH drugs according to the different classes of medication was different. There were more patients in the low persistence group who were discharged with traditional AH drugs (Beta-blockers and diuretics), and more patients were discharged with more than one AH drug class, compared with patients in the high persistence group. (Table 1).

### Risk Factors for Poor Outcomes after Stroke

The adjusted model showed that older age, history of stroke, myocardial infarction, atrial fibrillation, diabetes mellitus, and severe stroke increased significantly the risk of stroke recurrence, combined vascular events and death; while antiplatelet use significantly decreased the risk of these outcomes. The detailed ORs with 95% CIs of above risk factors are shown in Table 2.

**Table 2:** Risk factors for poor outcomes.

	Adjusted OR (95%CI)		
	Stroke recurrence	CVE	Death
Age			
≤65	Reference	Reference	Reference
66–75	1.42 (1.01–2.02)	1.56 (1.11–2.20)	2.14 (1.27–3.62)
≥76	2.17 (1.52–3.09)	2.56 (1.82–3.61)	4.68 (2.77–7.89)
History of stroke			
No	Reference	Reference	Reference
Yes	1.62 (1.44–1.82)	1.63 (1.46–1.82)	1.42 (1.23–1.65)
Myocardial infarction			
No	Reference	Reference	Reference
Yes	1.27 (1.10–1.48)	1.52 (1.32–1.75)	1.04 (0.86–1.25)
Atrial fibrillation			
No	Reference	Reference	Reference
Yes	1.50 (1.25–1.79)	2.41 (2.04–2.85)	2.14 (1.75–2.62)
Diabetes mellitus			
No	Reference	Reference	Reference
Yes	1.24 (1.10–1.41)	1.22 (1.08–1.37)	1.26 (1.08–1.48)
NIHSS scores at admission			
0–4	Reference	Reference	Reference
5–14	1.24 (1.08–1.41)	1.17 (1.02–1.31)	1.73 (1.45–2.06)
≥15	2.18 (1.83–2.60)	2.92 (2.47–3.45)	7.62 (6.24–9.31)
Antiplatelet agents use at discharge			
No	Reference	Reference	Reference
Yes	0.73 (0.65–0.83)	0.59 (0.53–0.67)	0.30 (0.26–0.35)

Abbreviations: CVE, Combined vascular events; CI, confidence interval; OR, odds ratio.

### Impact of Persistence with AH Drugs on Stroke Outcomes

Unadjusted analyses revealed that those with high persistence (≥75%) with AH drugs were less likely to experience an outcome than those with low persistence (stroke recurrence: 13.7% vs. 20.0%, p<0.0001; combined vascular events: 16.3% vs. 26.3%, p<0.0001; death: 5.8% vs. 18.4%, p<0.0001) (Table 3). After adjusting for potential confounders, the multivarible analysis revealed that high persistence (≥75%) with AH drugs was associated with a decreased risk of stroke recurrence (odds ratio, 0.78; 95% CI, 0.68–0.89), combined vascular events (0.71; 0.63–0.81) and death (0.44; 0.36–0.53) compared with low persistence. (Table 3).

**Table 3:** Event rates and odds ratios of stroke outcomes at 1 year for IS patients with hypertension.

		OR (95% CI)	
Outcomes at 1 year	Event rates	Crude	Adjusted*
**Stroke recurrence**			
Low persistence (<75%)	1150 (20.0%)	Reference	Reference
High persistence (≥75%)	364 (13.7%)	0.63 (0.56–0.72)	0.78 (0.68–0.89)
**Combined vascular events**			
Low persistence (<75%)	1510 (26.3%)	Reference	Reference
High persistence (≥75%)	433(16.3%)	0.55 (0.49–0.62)	0.71 (0.63–0.81)
**Death**			
Low persistence (<75%)	1056 (18.4%)	Reference	Reference
High persistence (≥75%)	154 (5.8%)	0.27 (0.23–0.33)	0.44 (0.36–0.53)

Abbreviations: OR, odds ratio; CI, confidence interval.

* Adjusted for age, level of education, monthly household income, marital status, history of stroke, myocardial infarction, atrial fibrillation, diabetes mellitus, antihypertensive medication history, class of prescribed antihypertensive drug at discharge, severity of stroke on admission, dysphagia, co-medication at discharge (antiplatelet agents, anticoagulants, lipid-lowering agents and anti-diabetic medications).

## Discussion

The results from our large and prospective study, conducted in China, showed that high persistence with AH therapy was associated with a lower risk of an adverse clinical outcome (death, combined vascular events, or recurrent stroke) among patients with IS and known to be hypertensive treated in routine clinical settings.

To our knowledge, this is the first study to investigate the association between AH persistence and clinical outcomes among IS patients for secondary prevention. Although Ovbiagele et al [15] reported that adherence to medication as secondary prevention for stroke was associated with positive stroke outcomes. Notably, that study measured adherence to non-specific pill prescription, rather than adherence to AH therapy. Moreover, Kettani et al [16] found that patients with high adherence to AH therapy (≥80% calculated using the medication possession ratio) had a significantly lower risk of cerebrovascular disease (OR 0.78, 95% CI 0.70 to 0.87), compared with patients with lower adherence. In contrast to our study, patients in this study were taking AH therapy as primary prevention for stroke. Although the Adherence eValuation After Ischemic stroke–Longitudinal (AVAIL) study [17] focused on secondary preventive medication persistence and adherence after stroke, no results about the association between medication adherence and stroke outcomes were published to date.

Apart from improved blood pressure control, a number of other reasons could be proposed to explain the association between high adherence to AH therapy and positive stroke outcomes. For example, patients who were at lower risk of death at 12 months may have had a milder initial stroke. In addition, it is possible that patients who report adherence to one medication may be more likely to adhere to other recommended treatments. In this study, patients with high adherence to AH therapy may have also adhered to other prescribed therapies, including antiplatelet agents, anticoagulants, lipid-lowering agents and anti-diabetic medications. However, after adjustment for all covariates (including severity of stroke and the use of above secondary medication), significant positive associations between persistence with AH therapy and IS outcomes still existed.

We recognize that our study does have limitations. Persistence was measured by patient self-report, which may have led to an over-estimation of adherence rates. However, this is a simple and effective method to identify adherence with AH therapy. We also note that this method has been used for assessing medication adherence in several cardiac and stroke studies [18]–[20]. Adherence assessed by patient self-report has been shown to correlate well with adherence assessed by pill count or electronic medication monitors [21], [22]. Our study is also limited by the fact that blood pressure data during follow-up were unavailable, negating our ability to assess the effectiveness of the AH therapy. Fortunately, a recently published study demonstrated that AH medication adherence was positively associated with achieving target blood pressure [23].

In conclusion, the results from our study show that high persistence with AH therapy significantly decreased the risk of stroke recurrence, combined vascular events and death among IS patients. Our study reinforces the benefits of AH medications in routine clinical practice and highlights the importance of persistence with AH therapy among IS patients known to be hypertensive within the first year of an event.
